# Saunders’s Pocket Medical Formulary

**Published:** 1901-05

**Authors:** 


SAUNDERS'S POCKET MEDICAL FORMULARY. With an Appendix. By WILLIAM M. POWELL, M. D., Author of "Essentials of Diseases of Children." Sixth edition, thoroughly revised. Philadelphia: W. B. Saunders & Co. 1900. [Price, $2.00, net.]

That old standby, Saunders's Pocket Medical Formulary, as complete and useful as ever, is out for the sixth time, thoroughly revised. It has a thumb index, blank pages for additional formulæ, diet lists and dose tables, tables of incompatibles, tables of poisons and their antidotes, in short, a thousand and one pieces of useful information which the physician is ashamed not to possess, yet can't for the life of him remember. M. J. F.

